# Supplementary material for: Stepwise control of host–guest interaction using a coordination polymer gel
Source: Nat Commun. 2018 May 18;9:1987. doi: 10.1038/s41467-018-04303-8 (PMC5959896; doi:10.1038/s41467-018-04303-8)
Supplement: Supplementary file 1 — Supplementary Information [file 41467_2018_4303_MOESM1_ESM.pdf]

Stepwise control of host-guest interaction using a coordination polymer gel

Mukhopadhyay *et al.*

Supplementary Methods

Methods

Unless otherwise stated, all starting materials, reagents and dry solvents were purchased from commercial suppliers and used as received. Solvothermal reactions were carried out in a programmable Nabertherm air oven (TR-60). The water used in all the experiments was Millipore Milli Q grade (18 MΩ•cm). (*E*)-2-hydroxy-5-(phenyldiazenyl)benzoic acid (**AzSA**) was synthesized according to a reported procedure[1]. ^{1}H and ^{13}C NMR spectra were measured on a 500 MHz Bruker Avance DPX spectrometer using TMS and $CDCl_3$ as internal standards. Melting point was measured on a MEL-Temp-II melting point apparatus and is uncorrected. Electrospray ionization mass spectroscopy (ESI-MS) was performed on a Thermo Scientific Q Exactive Hybrid Quadrupole-Orbitrap electrospray ionization mass spectrometer. IR spectra were collected on a Perkin-Elmer Spectrum One FT-IR spectrometer using KBr (neat). Thermogravimetric (TGA) analysis was carried out using TA instruments Q 50, all samples were heated at 10 °C min^{-1} under N_2 atmosphere.

Synthesis of (*E*)-2-hydroxy-5-(phenyldiazenyl)benzoic Acid (AzSA)

Concentrated hydrochloric acid (20 mL) was added to an ice-cold solution of aniline (6.12 g, 65.7 mmol) and cooled to 0 °C. A cold (0–5 °C) solution of $NaNO_2$ (4.55 g, 65.7 mmol) was slowly added to the above solution. The resulting benzene diazonium chloride was then added dropwise to an alkaline solution of salicylic acid (9.15 g, 65.7 mmol) at pH 8-9 (7.0 g of NaOH, 175 mmol), to yield the corresponding hydroxy azobenzene carboxylic acid. The compound was filtered and recrystallized from 50% (v/v) ethanol-water mixture. The bright orange crystals obtained were collected by filtration, washed with water and dried in a vacuum oven. Yield: 11.93 g, 75%; m.p.: 218 °C; FT-IR (KBr): ν_{max} = 2850 (b), 1667 (s), 1615 (m), 1447 (m), 1484 (s) cm^{-1}; ^{1}H NMR (500 MHz, $CDCl_3$, TMS, 25 °C): δ = 10.92 (d, 4H, Ar-OH), 8.55 (d, 4H, Ar-H), 8.16 (d, 4H, Ar-H), 7.91 (d, 2H, Ar-H), 7.53 (t, 2H, Ar-H),

7.49 (d, 1H, Ar-H), 7.14 (d, 1H, Ar-H) ppm; ^{13}C NMR (125 MHz, $CDCl_3$, 25 °C): δ = 172.4, 164.4, 152.5, 145.5, 137.6, 130.9, 129.5, 129.1, 127.8, 122.8, 118.7 ppm; ESI-MS (m/z): $[M+Na]^+$ calculated for $C_{13}H_{10}N_2O_3$ = 265.07; found: 265.08.

Synthesis of Mg-CP

Mg-CP was prepared by modifying a reported procedure[2]. **AzSA** (1.0 g, 4.13 mmol) and $Mg(NO_3)_2{\bullet}6H_2O$ (1.75 g, 6.82 mmol) was dissolved in *N*,*N*-diethylformamide (DEF) (50 mL) by sonication inside a glass vial. The resulting orange solution was kept in a programmable air-oven and heated to 115 °C at a rate of 0.2 °C min^{-1}, and after 24 h, the temperature was slowly decreased (over a day) to 30 °C. The orange gel formed was purified by Soxhlet extraction using chloroform. In order to determine the critical gelation concentration, the concentration of **AzSA** in DEF was varied from 1-4 mg mL^{-1}. The magnesium salt was taken in appropriate ratio. Separate solvothermal reactions with different concentration of **AzSA** revealed that a nonflowing gel was observed only at a minimum concentration of 4 mg mL^{-1}, which was thus determined to be the critical gelation concentration. A yellowish-orange xerogel powder was obtained after drying the orange gel overnight in an air-oven at 80 °C and was used without any further purification. The formation of **Mg-CP** was confirmed by FT-IR, WAXS and ^1H NMR analyses and the coordination of **AzSA** with Mg^{2+} was confirmed by the absence of phenolic –OH peak as well as the shifts in the signals corresponding to the aromatic protons along with broadening of the ^1H NMR spectrum. Since both magnesium and zinc forms isoreticular **MOF-74** based structures, which shows similar X-ray diffraction pattern, we have carried out the room temperature synthesis of **Zn-CP**[3]. The $Zn(CH_3COO)_2{\bullet}2H_2O$ in DEF was added to **AzSA** in DEF and was stirred at room temperature. Precipitation was observed only after 30 min. The stirring was continued for 20 h and then the product was purified by repeated cycles of centrifugation and washing with DEF and methanol. The product obtained was dried in an oven and used for measuring WAXS data. The WAXS

pattern of control MOF, **Zn-CP** matched fairly with that of **Mg-CP** prepared under solvothermal conditions (Supplementary Fig. 1).

Preparation of Polyacrylamide Hydrogel

A 40% stock solution of acrylamide/bisacrylamide was prepared by adding 63 mL of deionized water to the commercially available acrylamide/bisacrylamide (19:1) powder mixture, dissolved the contents, filtered and stored at 4 °C. A 10% solution (100 mg mL^{-1}) of ammonium persulphate (APS) was prepared in deionized water and stored at -20 °C. Tetramethylethylenediamine (TEMED) was used as obtained commercially. For preparation of the hydrogel, 1.5 mL of the 40% stock solution of the monomer was diluted with 1.5 mL of deionized water and 300 μL of 10% APS solution was added. The resulting solution was vigorously whisked to prevent the formation of air bubbles. Subsequently 30 μL of TEMED solution was added to control the rate of polymerization process. The solution was left undisturbed for ~ 1 h in a glass petri dish or in a quartz cuvette to obtain a transparent hydrogel.

Preparation of Mg-CP⊃α-CD Trapped Polyacrylamide Hydrogel

750 μL of an aqueous solution of **Mg-CP** (2 mg mL^{-1}) was added to 750 μL of an aqueous solution of **α-CD** (58 mg mL^{-1}). To the resulting solution, 1.5 mL of 40% stock solution of acrylamide/bisacrylamide was added followed by 300 μL of 10% APS solution. The resulting mixture was vigorously whisked to prevent the formation of air bubbles. Subsequently 50 μL of TEMED solution was added to control the rate of the polymerization process. The solution was left undisturbed for ~1 h in a glass petri dish to obtain a transparent yellow hydrogel.

Optical Measurements

Solution state electronic absorption spectra were recorded on a Shimadzu UV-3600 scanning spectrophotometer equipped with a temperature-control system, using a 1 cm path length

quartz cuvette. Circular dichroism spectroscopy was performed on a JASCO-J-810 spectropolarimeter that was equipped with a JASCO PTC-423S Peltier-type temperature-control system. The studies were performed either on an aqueous solution or on a hydrogel in a quartz cuvette of 1 cm path length. A solution of **Mg-CP** in deionized water was taken in a 1 cm length quartz cuvette and its photoirradiation was carried out using a LOT-Oriel 200 W high pressure Hg lamp utilising $\lambda_{\text{band pass}}$ = 350 nm for about 2.5 h. For the titration of **AzSA**, addition of an ethanolic solution of **AzSA** to an aqueous solution of cyclodextrins (CDs) was avoided to prevent precipitation of the CDs, whereas in case of **Mg-CP**, a specific initial concentration of the different CDs was used. CD Binding studies were conducted by either adding increasing amounts of different cyclodextrins (c = 1.5 x 10^{-2} M) in water to a fixed concentration of **AzSA** (3 mL, c = 3.3 x 10^{-4} M) (Supplementary Fig. 6c) or by adding successive aliquots of **Mg-CP** (2 mg mL^{-1}) to a fixed concentration (3 mL, 1 x 10^{-3} M) of different CD solutions (Supplementary Fig. 7).

Morphological Analysis

Transmission electron microscopy (TEM) and high-resolution TEM with selected area electron diffraction (SAED) were performed on a FEI, TECNAI 30 G2 S-TWIN microscope with an accelerating voltage of 100 and 300 kV, respectively. TEM samples were prepared by dispersing 1 mg of the xerogel samples of **Mg-CP** in 5 mL methanol or water via ultra-sonication for 15 min and then dropcasting the solution on carbon coated copper grids. Images were obtained without staining.

Rheology Experiments

Rheological measurements were carried out in an Anton Paar Physical Modulated Compact Rheometre-150 Physica (Germany). A parallel plate sensor having a diameter 50 mm and a gap size of 0.05 mm was used. Measurements were carried out in amplitude sweep mode,

varying the amplitude gamma from 0.01-100 % log and keeping the angular frequency omega at 10 Hz.

X-ray Diffraction Measurements

WAXS measurements on **Mg-CP** and **Zn-CP** powder were carried out on a XEUSS 2D SAXS/WAXS system using a Genixmicrosource from Xenocs operated at 50 kV and 0.3 mA. The Cu K_α radiation (λ = 1.54 Å) was collimated with a FOX2D mirror and two pairs of scatter less slits from Xenocs.

Supplementary Figures

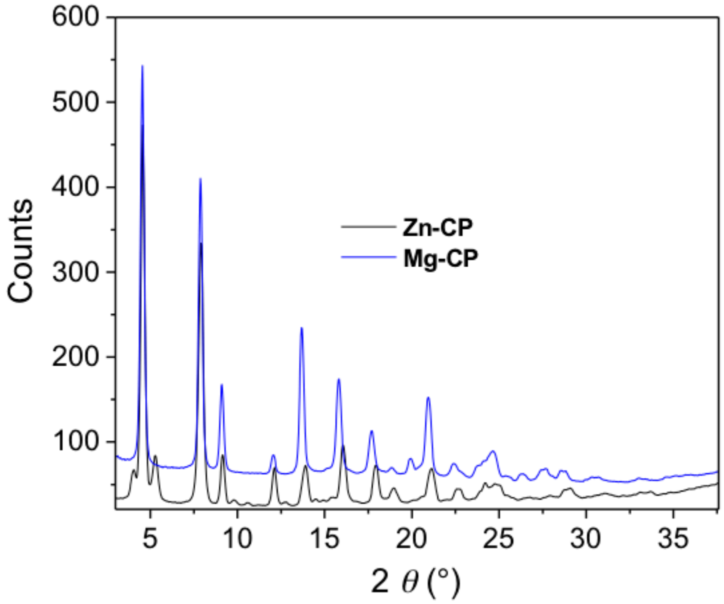

Supplementary Figure 1 | Structural comparison of Mg-CP with a control MOF (Zn-CP) having similar molecular connectivity. WAXS analysis of control MOF, **Zn-CP** (prepared under room temperature conditions) and **Mg-CP** (prepared under solvothermal conditions).

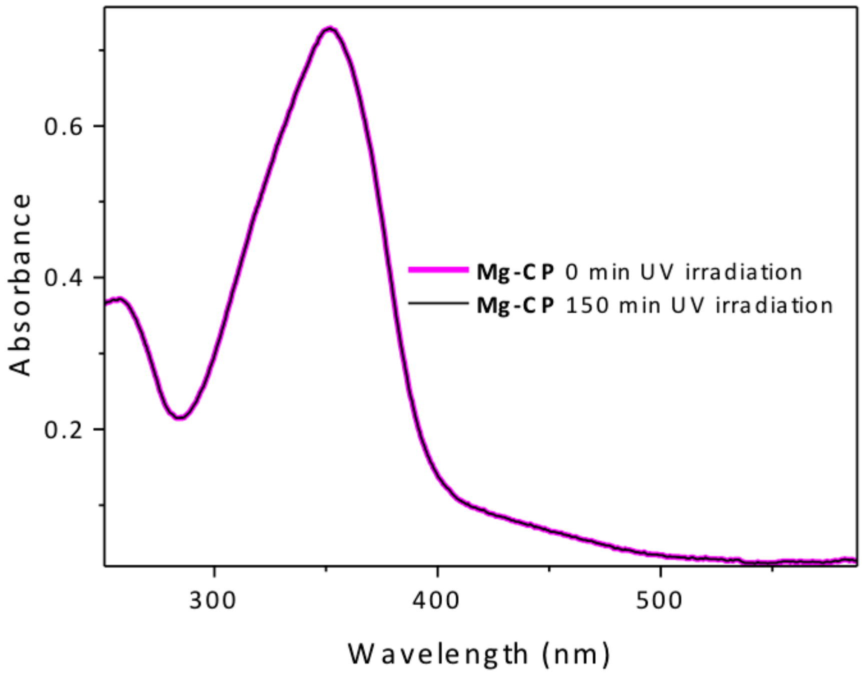

Supplementary Figure 2 | Effect of photoirradiation on Mg-CP monitored by UV-Vis absorption spectroscopy. Absorption spectra of a solution of **Mg-CP** (0.02 mg mL^{-1}) in water before and after 365 nm UV irradiation for 150 min.

a

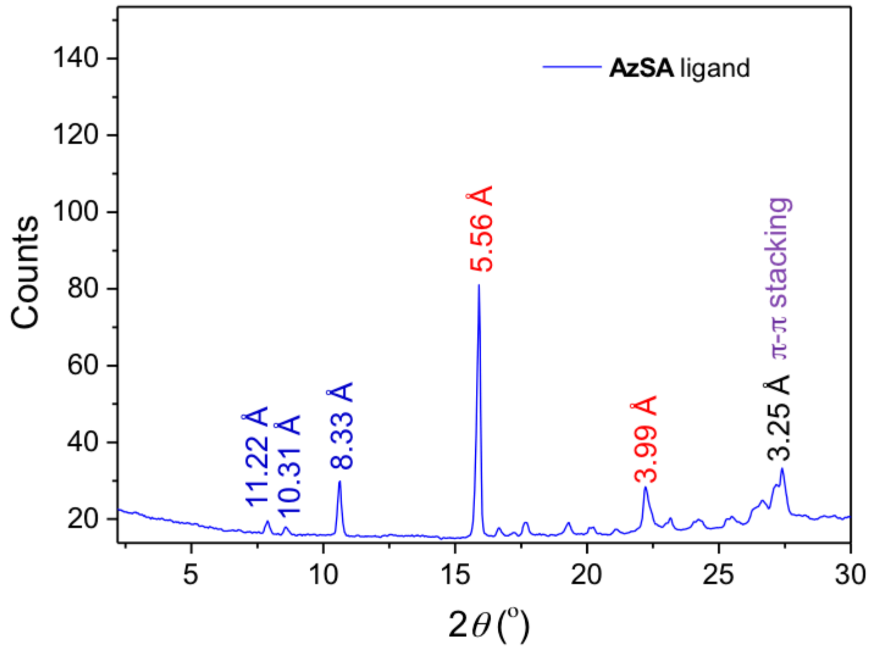

b

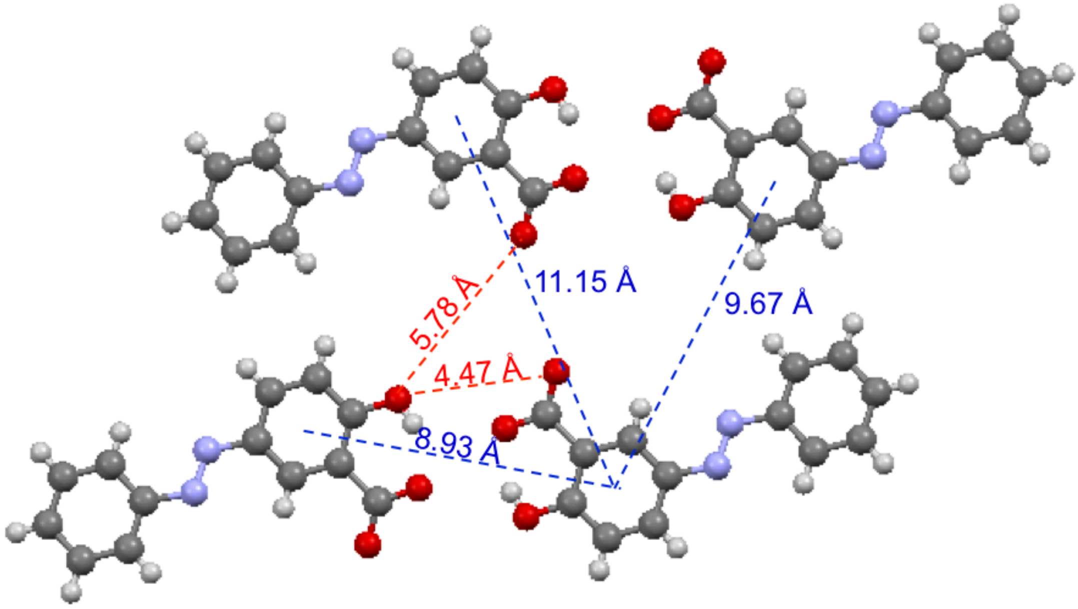

Supplementary Figure 3 | Structural analysis of AzSA. (**a**) WAXS analysis of **AZSA** showing the *d* spacing values corresponding to the diffraction peaks. (**b**) Proposed packing of **AzSA** based on the reported crystal structure of its ammonium salt[4]. Distance between the hydroxyl and carboxylic acid moieties and those between the phenyl rings are represented in red and blue colours, respectively.

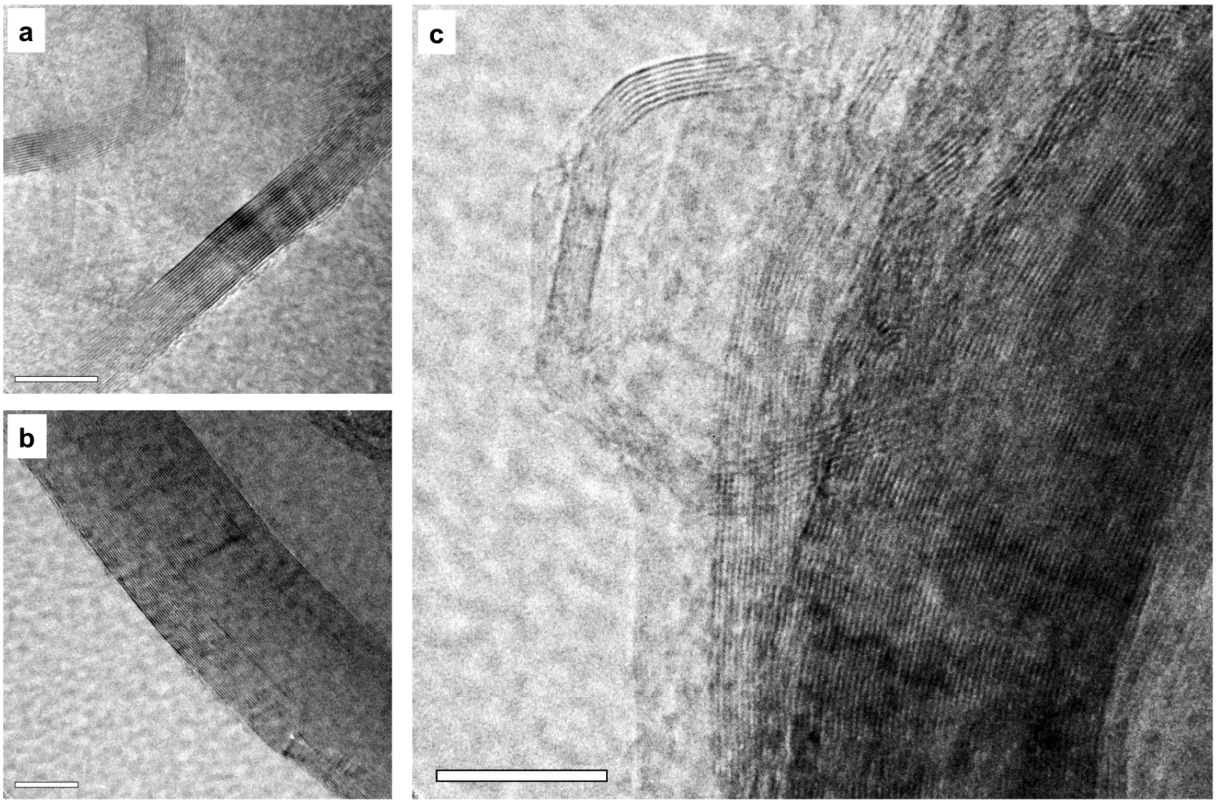

Supplementary Figure 4 | Morphological analysis of Mg-CP fibres by TEM. (**a**-**c**) High-resolution TEM images of **Mg-CP** fibres. Scale bar, 10 nm.

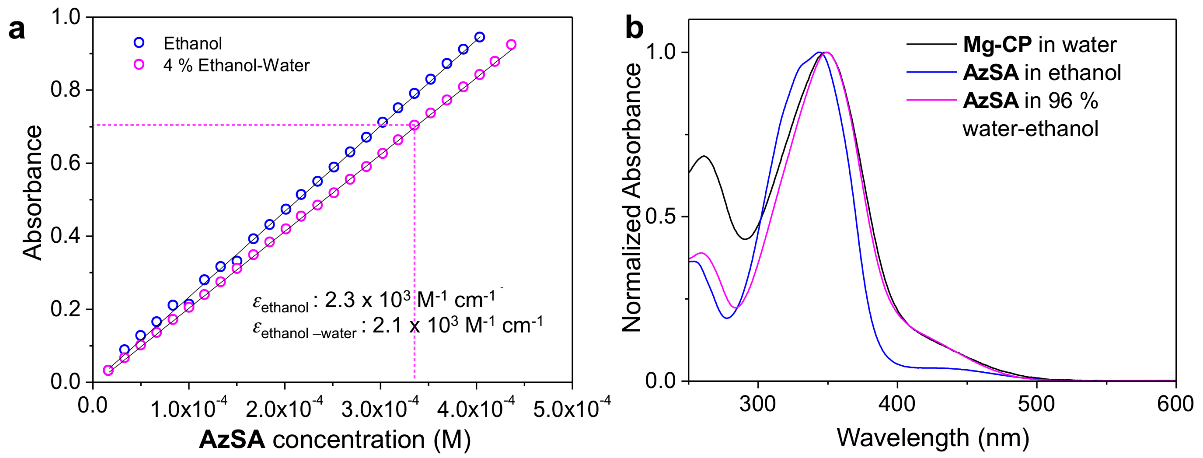

Supplementary Figure 5 | Calibration plot for the estimation of Mg-CP and AzSA in solution. (**a**) Calibration plot for **AzSA** in ethanol (blue) and 4% ethanol–water (magenta) obtained from concentration dependent UV-Vis absorption spectroscopy. Black lines indicate a linear fit to the observed data points. (**b**) Normalized absorption spectra of **AzSA** and **Mg-CP** in different aqueous solvents.

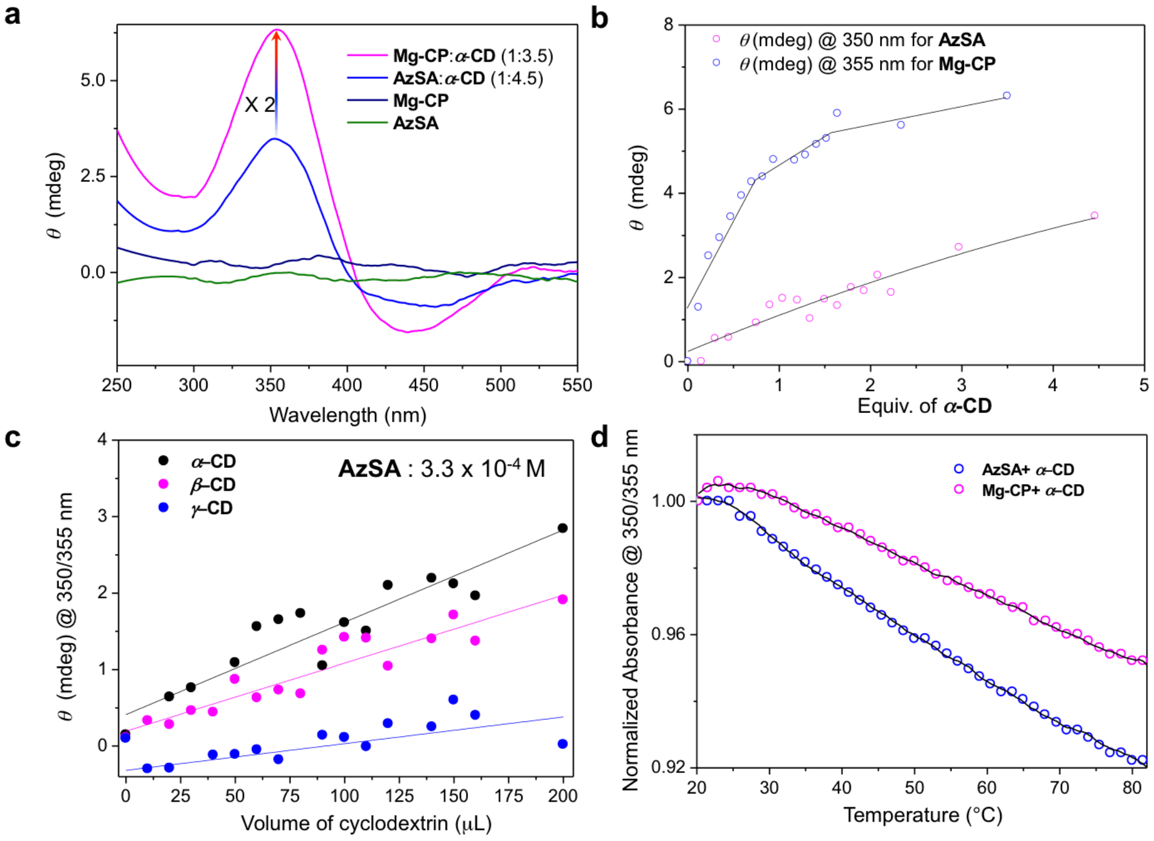

Supplementary Figure 6 | A comparative study on interaction of various cyclodextrins with AzSA and Mg-CP. (**a**) ICD spectra of **AzSA** and **Mg-CP** and those obtained upon addition of 4.5 and 3.5 equiv. of **α-CD**, respectively. The initial absorbance at 355 nm of both **AzSA** and **Mg-CP** solutions used were both fixed at 0.9 to maintain the same concentration. (**b**) Changes in the ICD signal (π–π* transition band) of **AzSA** and **Mg-CP** at different concentrations of **α-CD**. Black lines represent the corresponding non-linear fits to the observed data points. (**c**) Binding isotherms of **AzSA** with different types of cyclodextrins at 25 °C. Linear fit to the observed data points are also represented. (**d**) Thermal dissociation of **AzSA⊃α-CD** and **Mg-CP⊃α-CD** complexes monitored by UV-Vis absorption spectroscopy.

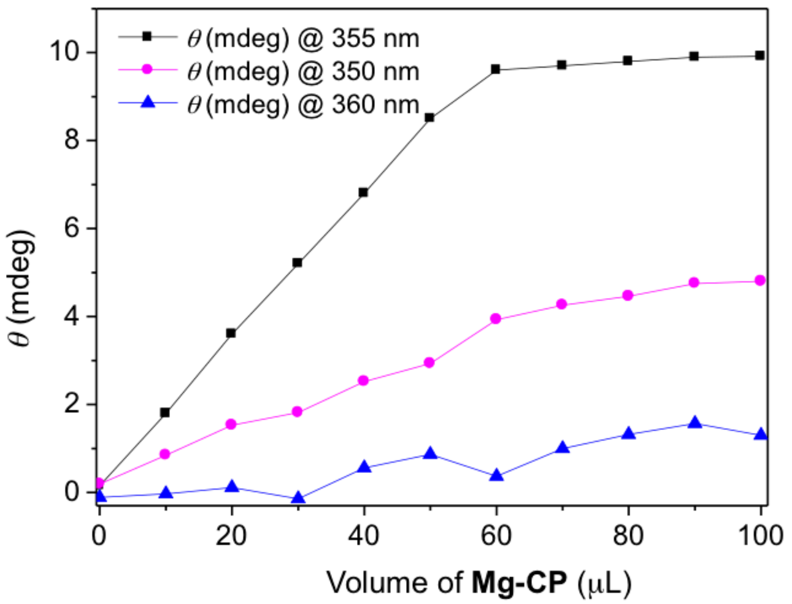

Supplementary Figure 7 | Interaction of Mg-CP with different types of cyclodextrins. Binding isotherms of **Mg-CP** with α**-CD** (black), β**-CD** (magenta) and γ**-CD** (blue) at 25 °C.

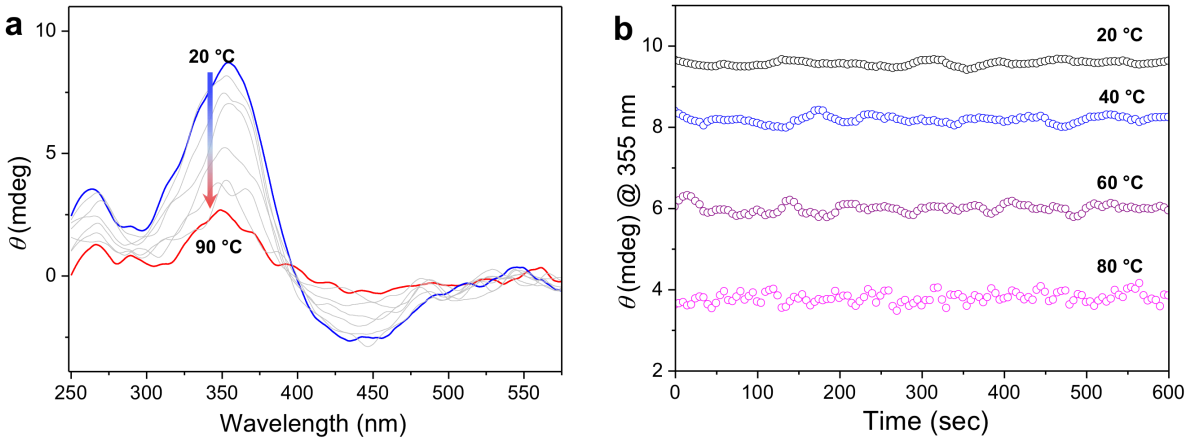

Supplementary Figure 8 | Thermoresponsive nature of AzSA⊃α-CD complex in solution. (**a**) Temperature dependent ICD spectra of **AzSA⊃α-CD**. Concentration of **AzSA** is 4.7 x 10^{-4} M. (**b**) Temperature controlled host-guest complexation of **AzSA⊃α-CD**. The ICD signal, which is a measure of the concentration of the formed host-guest complex, can be kept constant at a particular temperature.

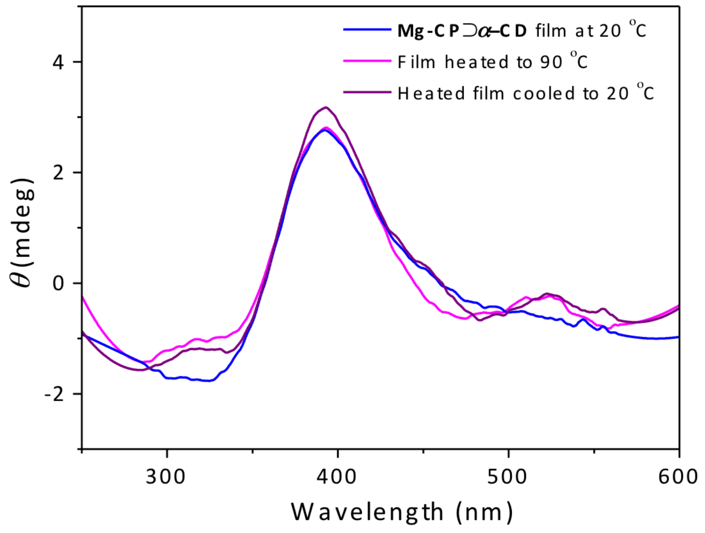

Supplementary Figure 9 | Thermoresponsive nature of Mg-CP⊃α-CD complex in film state. Temperature dependent changes in the ICD spectra of **Mg-CP⊃$\alpha-$CD** in the film state.

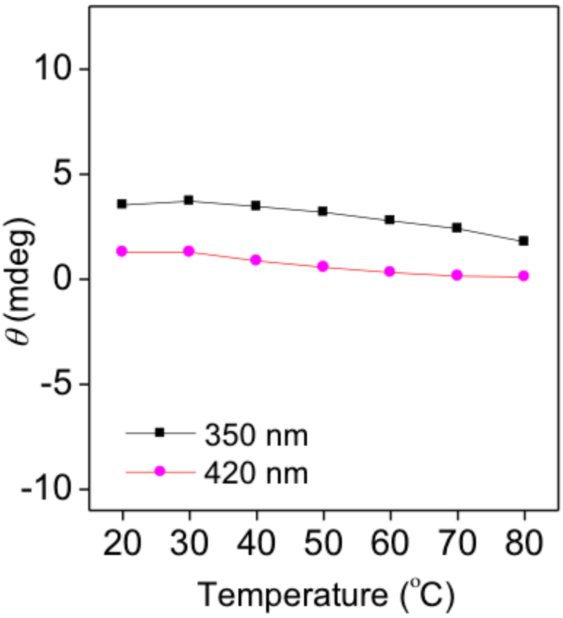

Supplementary Figure 10 | Control heating experiment with polymer hydrogel alone. Temperature dependent changes in the intensity of the CD band at 350 ($\pi-\pi^*$ transition band) and 420 nm ($n-\pi^*$ transition band) for the polyacrylamide hydrogel.

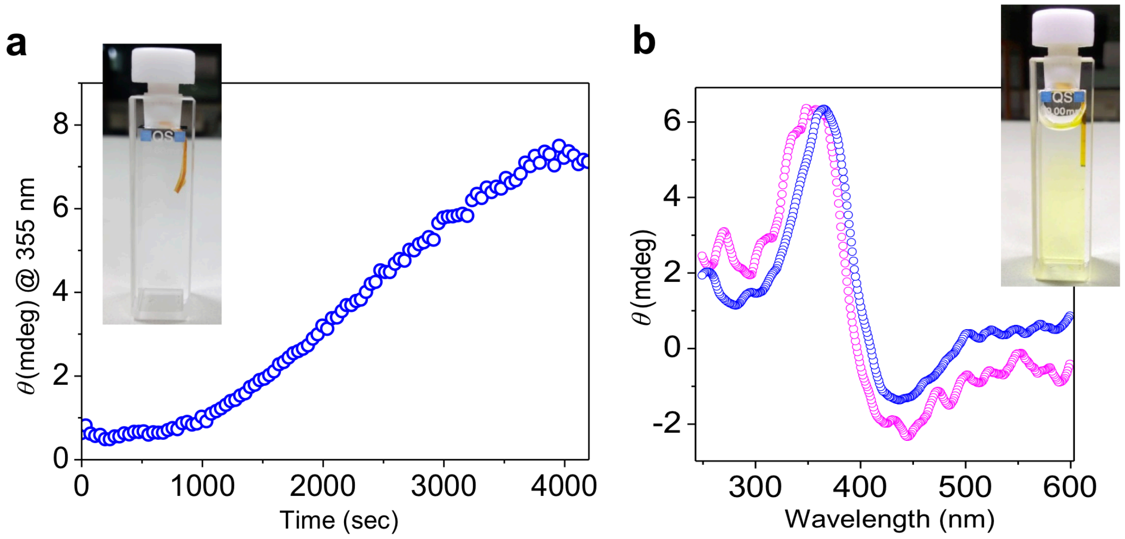

Supplementary Figure 11 | Stability studies on Mg-CP after being incorporated in hydrogel matrix. (**a**) Changes in the ICD signal at 355 nm corresponding to the gradual release of **Mg-CP**$\supset$$\alpha$**-CD** from hydrogel to water. Inset shows the photograph of the hydrogel strip fixed inside a quartz cuvette before the leaching experiment. (**b**) ICD spectra of the released **Mg-CP**$\supset$$\alpha$**-CD** complex in water (magenta). ICD spectrum of an aqueous solution of **Mg-CP**$\supset$$\alpha$**-CD** (blue) is provided for reference. Inset shows the photograph of the **Mg-CP**$\supset$$\alpha$**-CD** released into the solution after the leaching experiment.

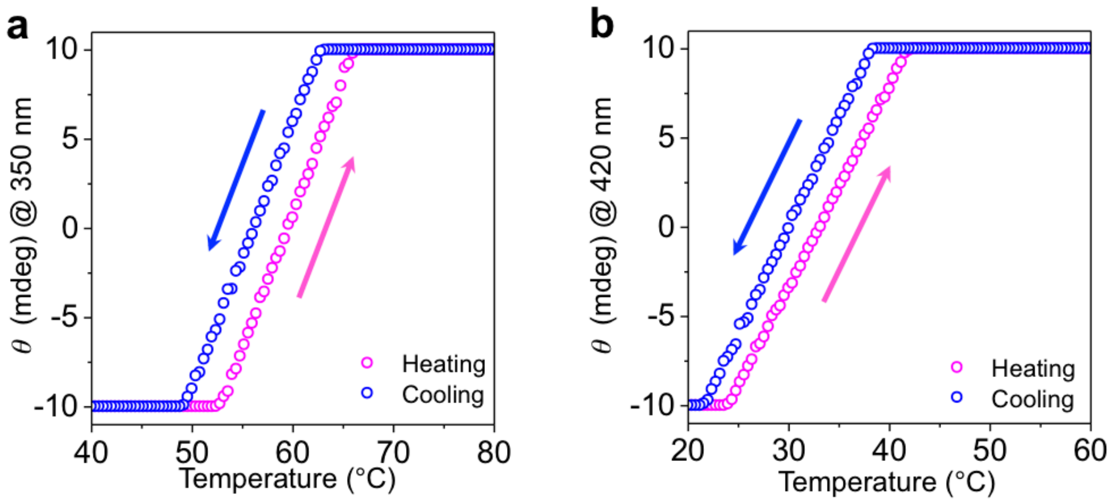

Supplementary Figure 12 | Heating experiment on Mg-CP⊃α-CD loaded polymer hydrogel. Temperature dependent changes in the ICD spectra of **Mg-CP⊃α-CD** entrapped in a hydrogel, recorded at (**a**) 350 and (**b**) 420 nm. Magenta and blue arrows represent heating and cooling cycles, respectively.

Supplementary References

1. Banerjee, I. A., Yu, L. & Matsui, H. Application of host-guest chemistry in nanotube-based device fabrication: photochemically controlled immobilization of azobenzene nanotubes on patterned α-CD monolayer/Au substrates via molecular recognition. *J. Am. Chem. Soc.* **125**, 9542-9543 (2003).
2. Brown, J. W. *et al.* Photophysical pore control in an azobenzene containing metal–organic framework. *Chem. Sci.* **4**, 2858-2864 (2013).
3. Díaz-García, M., Mayoral, A., Díaz, I. & Sánchez-Sánchez, M. Nanoscaled M-MOF-74 materials prepared at room temperature. *Cryst. Growth Des.* **14**, 2479-2487 (2014).
4. Yatsenko, A. V. & Paseshnichenko, K. A. *Syn* and *anti* conformations in 2-hydroxy-5-[(*E*)-(4-nitrophenyl)-diazenyl] benzoic acid and two related salts. *Acta Cryst.* **C70**, 493-497 (2014).
